# Harnessing Virtual Reality to Influence Attitudes Toward Beef Consumption: The Role of Empathy in Dietary Interventions

**DOI:** 10.3390/foods13233750

**Published:** 2024-11-22

**Authors:** Chia-I Hou, Jiun-Hao Wang, Kun-Sun Shiao, Che Cheng

**Affiliations:** Department of Bio-Industry Communication and Development, College of Bio-Resources and Agriculture, National Taiwan University, No. 1, Sec. 4, Roosevelt Rd., Taipei 106319, Taiwan; d00630002@ntu.edu.tw (C.-I.H.); d06227105@ntu.edu.tw (C.C.)

**Keywords:** meat consumption, food choice, health impact of foods, empathy, virtual reality

## Abstract

The excessive consumption of red meat, such as beef, is a growing global health concern linked to increased risks of cancer and cardiovascular diseases. The health consequences associated with red meat consumption were estimated to cost USD 285 billion globally in 2020, accounting for approximately 0.3% of total health expenditures that year. Understanding the psychological mechanisms behind food choices is crucial for changing consumption habits, fostering healthy behaviors, and achieving sustainable dietary patterns. To address these challenges, this study utilizes virtual reality (VR) as a persuasive tool to examine how empathy, as a psychological mechanism, influences the intention to reduce beef consumption and its impact on dietary attitudes. Using an experimental design with 142 participants, the study found that in the VR context, individuals with higher empathy scores experienced a stronger sense of presence, significantly influencing their attitudes toward beef consumption, mediated by the change in anti-beef-eating attitude (*p* = 0.029). This suggests that VR can serve as an effective medium to reduce individuals’ willingness to consume beef and consequently prevent health risks associated with excessive meat intake. This study also highlights the importance of considering individual empathy levels when designing VR interventions to maximize their effectiveness and promote healthier dietary habits, ultimately improving public health. However, one limitation of this study is that it only assessed short-term changes in attitudes following the VR intervention, without incorporating long-term follow-ups to determine if these changes are sustained over time.

## 1. Introduction

### 1.1. Background

Red meat (especially beef, lamb, and pork) is rich in nutrients, but scientific evidence shows that excessive consumption increases the risk of several cancers, such as pancreatic, colorectal, and stomach cancers, and can also lead to metabolic and cardiovascular diseases [1,2]. In 2020, health issues related to red meat consumption cost USD 285 billion globally, accounting for approximately 0.3% of total health expenditures that year [3].

Despite numerous public health campaigns encouraging people to reduce their red meat intake and increase vegetable consumption, these efforts have had limited success, as red meat still holds an important place in the diets of many countries. One reason for this limited success is that most people do not have the opportunity to see firsthand how animals are raised and processed into meat, such as in slaughterhouses or farms [4,5]. Instead, people usually only see the packaged meat products that are available in the market, leading to a disconnect that leaves consumers unaware of the reality of farm animals’ lives and deaths [6]. As a result, many people lack sufficient awareness of the health impacts of consuming meat [7].

“Seeing is believing” means that only what is witnessed firsthand can be considered credible [8]. As Ahn (2021) points out, direct experiences—where individuals have firsthand contact with an event—exert a stronger influence on attitude change than indirect experiences, where information is gained secondhand [9,10]. Direct experiences tend to form stronger attitudes, foster greater confidence in those attitudes, and lead to behaviors that are more consistent with the attitudes formed [9]. The findings of Fazio and Zanna (1981) and other research suggest that persuasive efforts should aim to emulate the characteristics of firsthand experiences to achieve maximum effectiveness [9,11,12]. Studies often compare virtual reality to traditional media like television or books, highlighting VR’s ability to immerse users in simulations that feel lifelike [9]. Herrera et al., (2018) also found that experiencing a perspective-taking narrative through a virtual environment led to greater empathy compared to reading the same narrative in text form [12]. Moreover, after discussion, Jeon et al., (2024) also suggested that VR effectively fosters empathy by providing immersive and interactive experiences, making it more engaging compared to traditional didactic or experiential training methods [13].

The emergence of virtual reality (VR) technology helps overcome the limitations of traditional health promotion methods by providing people with an opportunity to “see for themselves” the conditions of animals’ lives. This immersive experience can evoke stronger empathy and a sense of presence, thereby increasing the likelihood of changing attitudes [14].

While many studies have explored the use of VR technology to change food choices and promote environmentally friendly dietary behaviors, these studies have mainly focused on the impact of VR on dietary attitudes without delving into the role of the key variables involved in participants’ psychological processes. For example, Plechatá et al., tested the effectiveness of VR as a tool to promote pro-environmental dietary changes and found that VR interventions significantly reduced participants’ dietary carbon footprints while increasing the response efficacy and knowledge levels. However, their study did not consider psychological variables such as “presence” and “empathy” [15]. Similarly, other studies like those by Meijers, Song, and Fiore (2017), Xu et al., (2023), and Wan et al., (2022) investigated VR’s impact on dietary attitudes but did not address the psychological mechanisms [16,17,18].

To the best of our knowledge, only two studies have explored the relationship between VR, empathy, presence, and meat consumption attitudes. Of these, only one investigated the connection between VR, presence, empathy, and meat consumption attitudes in depth. Anderson et al., (2017) examined the effectiveness of VR in changing attitudes toward meat consumption (especially pork) and reducing animal suffering [19], while Herrewijn et al., (2021) studied VR’s role as a persuasive tool by enhancing empathy through an increased sense of presence [4].

While most studies suggest that a stronger sense of presence can increase empathy and thus influence attitude changes, not all studies agree that presence is the primary cause. Some studies propose that empathy itself may be the cause of presence. Specifically, individuals with greater empathy are more likely to feel a stronger sense of presence. For example, research by Sas (2004) and Nicovich et al., (2005) also found that empathy is a key factor determining whether someone can become immersed in VR. Those with greater empathy are more likely to emotionally connect with the virtual world, making it easier for them to experience immersion, reality, and belonging [20,21].

Although some studies have explored empathy’s potential to enhance the sense of presence in VR, few have delved into whether this indirectly affects VR’s overall effectiveness. If individuals with lower levels of empathy are less likely to be influenced by VR, this suggests a direct correlation between empathy and VR’s impact. If empathy is assumed to be generated by presence, then VR’s effect should be similar for individuals with different empathy levels. However, if greater empathy leads to a stronger sense of presence, VR’s effectiveness may depend on individual empathy levels.

Additionally, people often face an empathy gap when relating to abstract concepts and distant realities, such as the effects of meat consumption on animals and the environment. VR can bridge this gap by providing vivid, first-person perspectives of these realities, making abstract concepts more tangible and immediate, thus changing how people perceive meat consumption—from an abstract idea to a more concrete understanding of its impact.

### 1.2. Research Focuses

Based on the discussion above, our study has two primary research focuses:

First, we aim to provide participants with a first-person perspective, allowing them to witness an injured animal’s suffering rather than just seeing packaged meat products. We will explore how participants develop an understanding of the life and death of farm animals and examine the relationship between presence and empathy.

Second, this study aims to experimentally investigate whether differences in individual empathy levels alter the sense of presence experienced within virtual reality (VR) and, in turn, affect attitudes toward meat consumption. Given the rising health risks associated with excessive meat consumption, we are conducting this research to explore empathy’s influence on VR interventions.

To sum up, this study demonstrates how virtual reality (VR) can effectively reduce beef consumption by enhancing empathy, potentially mitigating health risks. It also highlights VR’s role as a health advocacy tool and emphasizes the importance of individual empathy in determining its effectiveness.

The primary contribution of this study lies in exploring how empathy, as a psychological mechanism, influences attitudes toward meat consumption through virtual reality (VR). Our findings reveal that individuals with higher empathy scores experience a stronger sense of presence in a VR setting, which significantly affects their attitudes toward beef consumption. Unlike previous studies that assume that presence precedes empathy, our results support the opposite view: empathy may be the key factor triggering a heightened sense of presence. Thus, our research not only demonstrates the potential of VR as an effective intervention tool but also emphasizes the importance of considering empathy as an individual trait when designing VR intervention strategies.

## 2. Literature Review

### 2.1. Empathy

Empathy is a complex, multidimensional psychological concept that involves the ability to understand and share the feelings and experiences of others [22]. Often described as the “vicarious and spontaneous sharing of affect” [23], empathy can be triggered by observing another person’s emotional state. This concept includes affective empathy, which refers to experiencing emotional resonance with others, and cognitive empathy, which involves understanding others’ perspectives [24,25,26,27,28]. These components enable individuals to connect deeply with others both emotionally and intellectually. Empathy is commonly regarded as a stable “trait” or “capacity”, meaning that some individuals display consistent empathic responses across different contexts, and this capacity remains relatively constant over time [29]. This view implies that while an individual’s sensitivity to others’ emotions and willingness to adopt others’ perspectives may vary, these differences are enduring aspects of a person’s character and are not easily influenced by situational factors.

#### 2.1.1. Cognitive Empathy and Affective Empathy

In studying how VR affects empathy, distinguishing between cognitive empathy and affective empathy is of significant importance.

Cognitive empathy and affective empathy are closely related but play different roles in emotion regulation. According to the study by Smith and Stamoulis (2023), cognitive empathy involves understanding others’ emotions from an objective perspective, which aids in emotion regulation and allows for the use of adaptive strategies such as reappraisal, thereby reducing emotional dysregulation. In contrast, affective empathy involves emotional responses to others’ emotions, which can amplify emotional reactions, making self-regulation of one’s emotions more challenging. Therefore, cognitive empathy helps in controlling emotions, while affective empathy may pose a challenge to effective emotion regulation [30].

In a study by Decety and Yoder (2016), the authors examined the relationship between individuals’ sensitivity to justice and the components of empathy, finding that affective empathy was not related to sensitivity justice sensitivity toward others. Instead, cognitive empathy and empathic concern predicted an individual’s sensitivity to justice for other people and promoted support for moral norms. This suggests that cognitive empathy plays a more significant role in fostering people’s concern for social justice [31].

Moreover, according to a study by Khanjani et al., (2015), there are significant differences in the performance of affective empathy and cognitive empathy across different age groups. As age increases, the level of affective empathy improves, while some aspects of cognitive empathy decline, which may explain why older individuals face challenges in interpreting emotional cues. These studies further demonstrate the different roles and changes in affective and cognitive empathy in individuals’ emotional responses and social functioning [32].

#### 2.1.2. Empathy, Compassion, and Sympathy

In addition to empathy, compassion and sympathy are also widely studied in psychology as sources of prosocial motivation. However, despite their similarities and occasional overlap with empathy, they differ significantly in terms of stability and context-dependence. Compassion, as defined by Goetz et al., (2010), is an emotional response that arises when an individual witnesses another person’s suffering and feels motivated to help. Unlike empathy, which is a stable trait, compassion is generally considered a temporary emotional state that fluctuates depending on the context, meaning that it is activated in response to specific situational cues and dissipates as those cues disappear. This variability distinguishes compassion from empathy, as compassion does not represent a consistent, ingrained characteristic [33].

Similarly, sympathy is a context-driven emotion characterized by feelings of concern and sorrow in response to recognizing another’s distress. Eisenberg et al. (1994) describe sympathy as an emotional response that arises from recognizing another individual’s emotional state or condition [34]. Sympathy can be triggered by a range of situations, from witnessing mild discomfort to observing severe suffering. Unlike empathy, which involves a deeper understanding and emotional resonance with others, sympathy does not necessarily require this in-depth understanding. It often arises as an instinctive reaction to another’s pain. Additionally, sympathy is frequently seen as a response that follows empathy, as it tends to emerge after an initial experience of empathy or affective sharing. Although sympathy and empathy may share certain emotional components, sympathy lacks the deeper understanding of another’s emotional state that is central to empathy [34,35].

These distinctions underscore fundamental differences between empathy, compassion, and sympathy, especially in terms of their stability and dependence on context. Generally, empathy is viewed as a long-term, stable trait that develops as individuals mature and become deeply embedded in their cognitive and emotional structure. Singer and Klimecki (2014) suggest that empathy, as an intrinsic characteristic, remains consistent across various contexts, allowing individuals to reliably display empathic behaviors. This stability allows empathy to serve as a reliable framework for understanding and connecting with others, regardless of changes in external conditions [36]. In contrast, compassion and sympathy are more immediate, context-dependent emotional responses. They are often short-lived, triggered by specific situations, and dissipate as the context changes. Condon and Barrett (2013) describe compassion as a “situated conceptualization”, meaning that its emotional experience is constructed in response to specific situational factors. Therefore, compassion and sympathy lack the enduring quality of empathy and are more susceptible to fluctuations based on the surrounding environment [37]. Barrett et al., (2014) support this view, suggesting that emotions like compassion are constructed and experienced differently depending on the immediate context, further emphasizing their variability and context-dependence [38].

Understanding empathy as a stable trait, and compassion and sympathy as context-driven emotions, is crucial to establishing the theoretical foundation of our hypothesis. By clarifying that empathy is a stable personal trait, while compassion and sympathy are context-dependent emotions, we provide a coherent and theoretically grounded basis for our hypothesis. Our hypothesis posits that participants’ inherent personal traits, such as empathy, will influence their sense of presence in virtual reality (VR), subsequently affecting their attitudes and behaviors. In other words, we assume that stable, enduring traits will consistently impact how participants perceive immersion in VR, regardless of specific situational contexts. This stable trait perspective suggests that empathy, as a deeply ingrained characteristic, will reliably affect participants’ perception of presence and thus influence their attitudes across various VR experiences.

However, if we assumed that presence in VR is primarily affected by context-driven emotions like compassion and sympathy, our hypothesis would face logical inconsistencies. Unlike empathy, compassion and sympathy do not provide a stable foundation for influencing presence, as they fluctuate based on situational cues. This would imply that participants’ sense of presence and subsequent attitudes may depend more on their immediate emotional state or the specific context of the VR experience, rather than on enduring personal characteristics. Such a context-driven assumption would not align with the aim of our study, which seeks to explore the role of stable, internal traits in shaping the VR experience. Instead, a focus on context-dependent emotions like compassion and sympathy would suggest that presence and attitudes are more susceptible to environmental factors than to stable personal traits, shifting the study’s emphasis away from intrinsic characteristics.

In conclusion, understanding empathy as a stable personal trait and compassion and sympathy as context-driven emotions clarifies the theoretical rationale for our hypothesis. The stability of empathy across different situations makes it a reliable predictor of presence and attitude changes in VR, while the fluctuating nature of compassion and sympathy makes them less suited for examining the long-term influence of personal characteristics. Therefore, our research is designed to investigate the effects of stable, internal traits in the VR experience, rather than the impact of transient, situational emotions.

### 2.2. VR and Empathy

Virtual reality (VR) refers to a technology that generates immersive, computer-created environments, enabling users to experience a sense of physical presence in both actual and fictional settings. This immersive experience is achieved through the use of VR headsets and other sensory devices that track user movements and provide sensory feedback, effectively transporting users to a different environment as though it is real [21,39].

Virtual reality (VR) technology is widely regarded as the “ultimate empathy machine”, with its effectiveness attributed to technological features that enhance users’ sense of “being there”. Schutte and Stilinović (2017) argued that presence provides a platform for experiencing empathy. Therefore, VR holds significant potential for cultivating empathy [40]. Barbot and Kaufman (2020) indicated that a strong sense of presence in a VR environment significantly enhances the ability to cultivate empathy, as presence deeply influences how authentically a user can connect with and process simulated experiences [14]. Although these VR research findings are contrary to our assumption that empathy leads to presence, they still offer valuable insights for reflection based on the literature.

By synthesizing these perspectives, empathy emerges as a complex, layered trait that can be nurtured through experiences combining cognitive understanding and emotional sharing. Advanced technologies like VR provide a unique platform for enhancing empathy, offering realistic, controlled environments that develop empathetic skills by simulating real-world complexities in a virtual format.

Drawing on the insights of several studies [27,39,40], empathy and virtual reality are discussed as follows: First, cognitive empathy involves the intellectual ability to identify and accurately understand another person’s thoughts and emotions [28,40]. VR technologies enhance this dimension by placing individuals in immersive scenarios that simulate others’ perspectives, thus fostering a better understanding of diverse viewpoints. Meanwhile, affective empathy refers to the emotional response triggered by the emotions of others, allowing one to feel what another person is feeling [41,42,43]. VR’s immersive nature can intensify these emotional connections, making the simulated experiences feel very real and immediate.

We have incorporated research cases into the discussion of empathy to differentiate between cognitive empathy and affective empathy. Below are some studies that can serve as examples to discuss the interaction between affective empathy, cognitive empathy, and VR.

Cummings et al., (2022) found that cognitive empathy is positively influenced by presence rather than self-location, contradicting some previous assumptions. Additionally, both self-location and presence have positive effects on affective empathy, suggesting that the more users feel that they are immersed in the story’s environment, the stronger their emotional response to the characters’ emotions is [44].

Bacca-Acosta et al.’s (2023) research presents the findings from a comparative study involving two virtual reality environments that portray stories about migrants in Colombia (South America). The study included 47 university students, who provided data through a self-reported questionnaire designed to measure emotional empathy, cognitive empathy, and other aspects. The results reveal that while virtual reality is effective in enhancing emotional empathy, it does not have a significant impact on cognitive empathy. This suggests that VR’s strength lies in evoking emotional responses rather than fostering a deeper understanding of others’ perspectives [45].

A paper by Jeon et al., (2024) discusses the use of VR for empathy education, specifically cognitive empathy. The VR game “Mysterious Museum”, discussed by Jeon et al., (2024), aims to foster cognitive empathy by helping users understand that individuals may perceive the same situation from varying perspectives [13]. The game involves ambiguous images and three-dimensional models that encourage users to see things from multiple perspectives, which is foundational for cognitive empathy. The study further emphasizes the effectiveness of VR in promoting empathy education because of its affordances, such as a sense of presence, which enhances both cognitive and affective empathy [13].

These findings actually show that the impact of VR depends on the design and context of its use. The studies by Cummings et al. and Jeon et al., focused on how VR content can effectively enhance cognitive empathy under specific conditions, such as games emphasizing perspective-taking or immersive news stories. These designs aim to encourage users to better understand others’ perspectives. On the other hand, the study by Bacca-Acosta et al. may not have designed VR experiences specifically for cognitive empathy, which might explain the lack of significant effects [13,44,45].

Therefore, the results of these three studies can be seen as complementary, highlighting different aspects of how VR influences empathy. Together, they indicate that VR is generally effective in enhancing affective empathy, but its impact on cognitive empathy depends more on specific designs and contexts.

However, we ultimately decided to use an integrated empathy scale rather than measure cognitive empathy and affective empathy separately.

This decision was based on several key considerations.

Firstly, using separate scales for cognitive and affective empathy increases the complexity of the measurement and burdens participants. Since these scales often contain a large number of items, they can lead to participant fatigue, which may affect the quality and reliability of the data. The use of an integrated scale simplifies the measurement process, reduces participants’ cognitive load, and increases their engagement in the measurement process [46].

Secondly, our research focuses on exploring the overall impact of VR on empathy rather than distinguishing between different types of empathy. Thus, using an integrated scale allows us to measure empathy as a holistic concept, which is more suitable for our research question. In addition, using an integrated empathy scale facilitates data analysis by providing a composite score, allowing us to directly associate VR experiences with overall changes in empathy during statistical analysis. This simplifies the separate analysis and discussion of cognitive and affective empathy, thereby improving the research efficiency. Furthermore, it contributes to model robustness by reducing data variability, making it easier to capture the effects of VR on empathy [47].

Cognitive and affective empathy often work together in real-life experiences, making it difficult to completely separate the two. In VR, participants may simultaneously feel the emotions of characters while understanding their situations and thoughts. Therefore, measuring these two dimensions as an integrated form of empathy can more authentically reflect participants’ comprehensive empathetic response in VR experiences, avoiding the oversight of possible interactions between the two [48].

The decision to use an integrated scale is also inspired by some previous research. Many studies have shown that the distinction between affective and cognitive empathy may be less pronounced in certain contexts, particularly in highly immersive environments. Thus, combining them into a broader empathy variable helps us better understand participants’ emotional and cognitive responses in VR experiences [29].

Another issue that is worth discussing is that the majority of previous empirical studies indicate that experiencing a stronger sense of presence through virtual reality can also trigger stronger empathy, leading participants to change their attitudes and behaviors, such as signing petitions and exhibiting prosocial attitudes.

Several studies are relevant to this issue. For example, Herrera et al., (2018) found that participants exposed to VR content were more likely to sign a petition related to the topic under investigation [12]. Bujić et al., (2020) similarly conducted a study to explore how media content presented using immersive worlds could influence attitudes toward human rights [49]. Based on the assumption that “stepping into another’s shoes” could trigger empathy, their experimental findings suggest that VR journalistic content can induce a positive shift in users’ human rights attitudes. Another example is Ahn et al.‘s (2013) experiment, which demonstrates that VR interventions, such as helping a colorblind person, can effectively elicit prosocial behavior in real life. Participants who experienced virtual colorblindness displayed more empathy and were more likely to offer help, demonstrating enhanced prosocial behavior due to a heightened sense of shared experience and empathy [50].

Despite the assumption in these influential studies that presence influences empathy, leading to changes in attitudes, they did not examine the potential confounding effect of empathy as a pre-existing ability. In this research, we explore the possibility that empathy, as an inherent ability, might lead to a heightened sense of presence.

As mentioned earlier, research by Nicovich et al., (2005) supports our argument by showing that an individual’s ability to empathize significantly influences their sense of presence in a computer-mediated communication (CMC) experience [21].

In an experiment conducted by Ling et al., (2013), participants were recruited to investigate how individual characteristics, such as empathy, influence the sense of presence in virtual environments. The experimental setup involved exposing participants to both a non-stereoscopic and a stereoscopic public speaking environment to assess their responses under different conditions. The results showed that empathy was significantly correlated with the Simulator Sickness Questionnaire (SSQ) [51].

Although virtual reality (VR) has been widely used to enhance empathy, research shows that its effectiveness is not always consistent. For instance, while VR can enhance emotional empathy, its impact on cognitive empathy is limited [52]. Furthermore, when the facial expressions or movements of virtual characters in VR lack realism, users may feel a sense of disconnection, reducing emotional engagement [53]. Some studies suggest that because VR cannot fully replicate real-life interpersonal interactions, and this simulation may not effectively elicit lasting emotional resonance [54]. In certain situations, users even respond to immersive technology with detachment or indifference, especially when the content is overly intense or unsettling [55]. Additionally, when the facial expressions and emotional cues in VR lack authenticity, it may also weaken empathy enhancement [40].

These studies indicate that due to challenges in technical limitations, the degree of immersion, and the realism of virtual characters [52,54], the effectiveness of VR in enhancing empathy is inconsistent.

We have designed an immersive VR scenario that allows participants to witness the suffering of animals, thereby enhancing empathy. By intensifying the sense of presence and realism of the scenario, we aim for participants to generate a direct emotional response. Although VR has shown limitations in previous empathy studies, this research provides a new perspective to examine the potential of VR in influencing attitude changes.

Based on previous research, this paper argues that presence in virtual reality is highly correlated with empathy; however, it challenges the common belief that presence leads to empathy. Instead, it suggests that empathy triggers a sense of presence.

Moreover, although we have already explained the rationale for choosing to measure empathy as a unified construct rather than separately assessing cognitive empathy and affective empathy, we believe that further exploration of the respective advantages and limitations of unified versus separate measurements remains a topic worthy of in-depth discussion. Therefore, we have elaborated on this point in the following sections to provide a more comprehensive explanation of our methodological choice.

#### Comparative Analysis of Empathy Measurement Approaches

When evaluating whether to measure cognitive empathy and affective empathy separately or use an integrated measurement, it is essential to compare these approaches based on conceptual differentiation, measurement efficiency, and applications in virtual environments. Below, an analysis of these two methods is presented based on insights from Caruso and Mayer (1998) [56], Davis (1983) [23], and Hogan (1969) [57].

Measuring cognitive and affective empathy separately allows for a clearer distinction between these two relatively independent constructs. Cognitive empathy typically refers to the ability to understand others’ feelings and perspectives, akin to theory of mind, whereas affective empathy involves emotional responses to others’ feelings. Measuring these two components separately can more accurately capture the unique characteristics and impacts of each construct [23,56].

For example, Davis’ multidimensional empathy scale employs different subscales to measure perspective-taking (cognitive dimension) and empathic concern (affective dimension), allowing for a more detailed analysis of distinct aspects. In contrast, Hogan’s empathy scale primarily focuses on cognitive empathy, emphasizing social function and role-playing abilities [57].

On the other hand, integrated measurement has the advantage of being more efficient, simplifying the measurement process. Caruso and Mayer (1998) pointed out that an integrated empathy measure can assess multiple constructs simultaneously, thereby reducing participant fatigue and improving data collection efficiency [56]. However, integrating two constructs into a single measurement may obscure subtle differences between them, which can be particularly relevant when studying specific interactions between cognitive and affective empathy. It may then be challenging to distinguish which empathy dimension influences certain behaviors or scenarios.

When exploring the relationship between empathy and presence in virtual environments, separate measurements of cognitive and affective empathy can help identify which component has a greater impact on participants’ sense of presence. For instance, cognitive empathy may emphasize role-playing and understanding storylines, while affective empathy is more related to emotional resonance. Thus, separate measurement may enhance our understanding of empathy’s impact on presence in virtual environments.

In virtual reality (VR) and empathy research, the trend leans towards using a single or integrated empathy measurement instead of separating cognitive and affective empathy within an experiment. This choice simplifies the experimental design and is better suited for observing overall empathy responses, particularly when the research goal is not to focus specifically on the distinct effects of cognitive or affective empathy. For instance, van Loon et al., (2018) used a single indicator to measure cognitive empathy, demonstrating the feasibility of treating empathy as a unified construct, particularly in studies requiring an overall assessment of empathy responses [58].

Additionally, Shin (2018) explored how VR can evoke empathy and embodied experiences, concluding that VR effectively stimulates participants’ empathy, particularly when presenting highly emotional scenes [39]. This finding supports the rationale for employing an integrated measurement in VR research, as it can capture the overall emotional response.

Hamilton-Giachritsis et al.’s study (2018) also utilized a unified measurement approach. Their study used immersive virtual reality to simulate the experience of being a child, allowing mothers to assess its effects on their perspective-taking and empathy [59]. The unified measurement approach was effective in evaluating participants’ cognitive and emotional responses, demonstrating that this method is feasible for studying the impact of empathy in VR environments [59].

Regarding the suggestion to conduct a comparative analysis of separate versus integrated measurements, such research could yield valuable insights into empathy measurement and its applications in virtual environments. For example, Herrera et al., (2018) found that VR can elicit strong emotional resonance in the short term, suggesting that an integrated measurement is useful in such contexts [12]. However, if the goal is to understand the distinct effects of cognitive and affective empathy in specific contexts, a separate measurement may be more beneficial.

The use of an integrated empathy scale strikes a balance between efficiency and effectiveness, particularly suitable for capturing overall empathy responses in complex environments such as VR. However, if future research aims to explore specific aspects of cognitive or affective empathy, or to study their interactions in virtual environments, then a separate measurement approach would be preferable.

Overall, research involving VR and empathy increasingly favors integrated empathy measurement due to its holistic perspective, which facilitates a better understanding of how VR influences users’ emotional and cognitive responses in complex social contexts. These studies contribute to advancing our understanding of VR’s potential to foster positive social behaviors.

### 2.3. Empathy Toward Humans and Animals

Research on empathy towards humans and animals has garnered considerable attention, revealing significant insights into the interconnectedness of these empathic responses. Taylor and Signal (2005) investigated the relationship between human empathy and attitudes towards animals, finding that individuals with higher levels of empathy towards humans are also more likely to exhibit empathetic attitudes towards animals. Their study underscores the role of empathy as a fundamental component in shaping human perceptions and treatment of animals, highlighting the moral and ethical implications of human–animal interactions [60].

Westbury and Neumann (2008) examined empathy-related responses to film stimuli depicting both humans and non-human animals in distressing situations. They discovered significant linear trends indicating that stimuli that are closer in phylogenetic relatedness to humans elicited higher empathy ratings and stronger physiological responses, such as corrugator Electromyography (EMG) activity and phasic skin conductance responses (SCRs). These findings suggest that empathic responses towards humans can generalize to other species, with stronger responses for species that are more similar to humans, supporting the notion that human empathy can extend beyond species boundaries [61].

Paul (2000) also explored the link between empathy for humans and animals, demonstrating a significant relationship between the two. His research indicates that individuals with high levels of empathy towards humans are likely to show similar levels of empathy towards animals [62]. This supports the idea that empathy is a broad, inclusive trait influencing responses to both human and non-human suffering.

Collectively, these studies highlight a significant overlap in empathetic responses towards humans and animals. Empathy is not confined to intraspecies interactions but extends to interspecies relations. Higher empathy levels towards humans often correlate with greater empathy towards animals, suggesting a generalized capacity for compassion that transcends species boundaries.

Given these findings on empathy’s extension to animals, it is plausible that individuals with higher empathy levels are not only more likely to adjust their dietary habits but also exhibit more negative attitudes toward beef consumption due to concerns about animal suffering. Empathetic responses towards animals, especially when witnessing their conditions in farming environments, may foster negative attitudes toward practices that cause harm to these animals. This, in turn, could lead to a reduction in beef consumption as individuals reassess their dietary behaviors based on their attitudes. Thus, these insights are important for understanding how empathy-based interventions, including VR experiences that highlight animal suffering, can effectively influence consumer attitudes and behaviors related to meat consumption.

### 2.4. Presence

VR is a technology that creates an immersive, interactive environment that is capable of simulating real or imagined worlds. At the heart of VR is the computer-generated space that creates a sense of presence, making users feel as if they are in a different location [18]. The concept of presence denotes the sensation of being physically “there” within a virtual environment [6,63,64,65,66,67] and is a key element that distinguishes VR from other forms of media.

Lombard and Jones (2015) provide a detailed definition of presence, characterizing it as a psychological state where individuals perceive virtual objects as real, encompassing both sensory and nonsensory experiences [68]. This definition underscores the subjective nature of presence [39], which can vary significantly among users depending on their engagement and the quality of the VR environment.

Lombard and Ditton (1997) define presence as a perceptual illusion of non-mediation, in which users experience a sense of being immersed in the virtual environment rather than merely observing it [69]. This immersive experience is achieved through sensory inputs that engage the user’s vision, hearing, and sometimes touch, providing a holistic and engaging experience [4].

Slater and Steed (2000) further explore this concept by introducing the idea of a “virtual presence counter”, which measures the level of presence experienced by users in VR environments. Their research highlights the importance of real-time responses within the virtual space, which significantly enhance the user’s sense of presence. Previous research comparing the effects of content experienced in VR versus non-VR formats (e.g., video, text) has consistently demonstrated that participants in more immersive conditions report experiencing higher levels of presence in the virtual world than in other formats, such as video or text [65].

In conclusion, VR serves as an effective medium for crafting immersive experiences by cultivating a profound sense of presence, which is influenced by both the design of the VR environment and the user’s interaction with the virtual world.

### 2.5. VR, Dietary Choices, and Meat Consumption

Many studies have explored how virtual reality (VR) technology can be used to influence food choices and promote pro-environmental dietary behaviors and attitudes. Nevertheless, the majority of these studies have concentrated on examining the general effects of VR on dietary behaviors and attitudes, often without investigating key psychological factors like “presence” and “empathy”. These variables are crucial in understanding the underlying psychological processes that contribute to behavior and attitude change.

For example, Plechatá et al., (2022) evaluated the effectiveness of VR for encouraging pro-environmental dietary behaviors and attitudes. Their findings indicated that VR interventions significantly reduced participants’ dietary carbon footprints and increased their response efficacy and knowledge. However, the role of psychological variables such as “presence” and “empathy” was not examined in their research [15].

Similarly, Meijers et al., (2022) used VR technology to promote sustainable food choices by focusing on environmental and health messages. Despite showing positive effects, the study did not explore the specific influence of participants’ sense of immersion and emotional connection in changing behaviors and attitudes [16]. Song and Fiore (2017) proposed guidelines for designing and assessing VR environments for healthy eating behaviors and attitudes, but these guidelines did not highlight the importance of empathy and presence as influential psychological factors [70].

Several other studies also lacked a focus on empathy and presence. For instance, Xu et al., (2023) studied consumer meat choices in virtual supermarkets using VR [17], and Wan et al., (2022) examined how VR could be used to promote sustainable food choices through visual design (e.g., color contrast) [18]. Furthermore, Smit et al., (2021) explored the application of VR to encourage healthy eating habits in children [71], whereas Crofton et al., (2021) examined the sensory experiences of food products within immersive VR environments [72]. However, none of these studies examined the role of empathy and presence during these experiences

Despite the promise of VR in terms of influencing dietary behaviors and attitudes, a gap exists regarding the specific roles of empathy and presence in this process. Our study seeks to fill this gap by focusing on how empathy and presence can function as psychological mechanisms to amplify the effects of VR on dietary behaviors and attitudes, particularly in reducing meat consumption. By building on previous studies and examining these key variables, we aim to provide more nuanced insights into how VR can effectively influence food-related behaviors and attitudes. This approach also aligns with the need for further exploration of how previous studies have shaped the current research, highlighting the importance of understanding empathy and presence in VR-based interventions.

Furthermore, some studies have investigated VR’s specific influence on meat consumption. Anderson et al., (2017) conducted an experiment integrating VR into traditional media approaches to explore its impact on consumer attitudes and behaviors toward pork consumption. In their study, participants were immersed in VR experiences that simulated different scenarios, including highlighting the health benefits of pork or emphasizing its ethical and environmental repercussions. Their results suggested that VR could significantly amplify the impact of the content, with scenarios depicting negative aspects of pork production resulting in a considerable decrease in consumption intentions. This research underscores the role of empathy in motivating individuals to reduce meat consumption and reflects the potential of VR to influence broader dietary behaviors and attitudes [19]. Fonseca and Kraus (2016) also explored the impact of VR on meat consumption. They found that participants using head-mounted displays (HMDs) reported greater changes in their consumption intentions compared to those using hand-held displays, highlighting the more immersive nature of HMDs. The emotional engagement fostered by immersive VR experiences made the benefits of plant-based diets more tangible, thus motivating participants to change their eating habits [73].

Another study by Herrewijn et al., (2021) demonstrated that VR could enhance empathic concern and promote intentions to reduce meat consumption, especially when emphasizing animal suffering. Their findings showed that VR generated higher levels of empathy than traditional video, with presence acting as a mediator. However, our current study aims to delve deeper into the relationship between these factors, questioning whether empathy directly enhances presence, and thereby further impacting the efficacy of VR interventions [4].

In conclusion, VR has a unique capacity to evoke strong emotional responses—particularly by making the suffering of animals and the environmental impact of meat consumption more vivid through immersive experiences. This emotional engagement fosters empathy and effectively influences attitudes, contributing to reduced meat consumption. By understanding these mechanisms, our study seeks to advance the field by clarifying the psychological processes that make VR an effective tool for influencing dietary behaviors and attitudes and encouraging sustainable choices.

### 2.6. Individualistic Differences

Do all individuals experience the same level of presence, irrespective of varying factors? Individual differences in experienced presence have garnered increasing attention from researchers [74]. Given that presence is as much about psychological factors as it is about perception, it naturally follows that some people are better at achieving a sense of presence than others, similarly to how some people are more empathetic than others. Nicovich et al.’s research indicates that an individual’s ability to empathize significantly affects the level of presence that they experience in computer-mediated communication (CMC) environments [21]. Makransky and Petersen (2021) posit that individual variations in perception contribute to differences in presence within virtual environments [74]. This suggests that even within identical virtual experiences, different individuals may perceive presence differently, influenced by factors such as variations in attentional capacities.

Sacau, A., Laarni, J. and Hartmann, T. (2008), experimentally confirmed that an individual’s pre-existing interest in the content of a virtual world can increase their attention and influence resource allocation, thereby enhancing their experience of presence [75].

Ling et al., (2013) explored how variations in individual characteristics such as personality traits, empathy, and cognitive styles might influence differences in perceived presence within a virtual environment designed for public speaking. The experimental setup involved exposing participants to both a non-stereoscopic and a stereoscopic public speaking environment to assess their responses under different conditions. The results only showed that empathy was found to be significantly correlated with the Simulator Sickness Questionnaire (SSQ) [51].

However, while Nicovich et al.’s experiment involved participants learning to fly a light plane, and Ling et al., concentrated on public speaking in virtual reality, this study distinguishes itself by focusing specifically on the role of empathy within virtual environments [21]. Unlike earlier research, which primarily examined presence through task-based or skill-oriented activities, we aim to explore how individual differences in empathy influence the sense of presence in virtual worlds. This novel approach seeks to illuminate the emotional and psychological mechanisms underlying presence, offering a deeper understanding of how empathy uniquely interacts with immersive experiences.

### 2.7. Emotional Biases and Meat Consumption

In consumer psychology research, the impact of emotional bias on choice is an important but still underexplored topic. However, the research on the influence of emotional bias on consumer attitudes and decision-making remains relatively limited. Although emotional bias may play a role in the consumer decision-making process, systematic research in this area is still relatively scarce. Some studies use “emotional bias” in their titles but do not explicitly or accurately address the concept of “emotional bias” in their research. There are studies that mention “emotional bias” in the title, but in the main text, they often use terms like “emotional stimuli”, “emotional components”, or even “emotional lability” instead, making the concept of emotional bias unclear. For example, in a study by Murphy et al., (1999), the text only uses “emotional stimuli” to describe emotional responses, without thoroughly discussing the meaning of emotional bias [76].

Similarly, Magai et al., (2000) mention the relationship between adult attachment styles and emotional biases in their research but do not provide a clear definition of emotional bias in the text, instead describing emotional responses as variables [77]. Likewise, Novianggie and Asandimitra (2019) mention the influence of behavioral bias, cognitive bias, and emotional bias on investment decisions, but the discussion lacks a detailed distinction between these biases, especially without a specific definition of emotional bias [78]. These examples show that, although emotional bias is an important concept, its meaning has not been adequately explained or elaborated upon in many studies.

Despite the relatively limited research in this field, based on our literature review, to the best of our knowledge, Yuan et al., (2019) and Pompian’s (2011) studies are the only two studies that systematically discuss emotional bias. Yuan et al., (2019) further demonstrated through a meta-analysis that emotional bias varies with stimulus type, arousal level, and task setting [79,80]. Pompian [80] explored the influence of various emotional biases on decision-making, and we therefore chose to use the studies by Yuan et al. and Pompian as a basis, building on their conclusions to infer the potential impact of emotional bias on meat consumption choices [79,80]. Below, we discuss these two studies separately.

#### 2.7.1. Negative Bias and Positive Bias

According to Yuan et al., (2019), emotional bias is considered an important evolutionary outcome of human responses to emotional stimuli. These biases have played a crucial role in survival, driving us to pursue rewards and avoid threats, thus enhancing our adaptability to the environment [79]. Although emotional stimuli are generally divided into positive and negative categories [81], human responses to these stimuli are often asymmetrical due to the different survival values that are inherent to each emotion.

Specifically, emotional bias manifests in two main phenomena: negative bias and positive bias. Negative bias refers to the tendency for people to respond more strongly to negative stimuli than to positive stimuli [82]. This heightened sensitivity to negative events or information significantly impacts cognition, attention, and decision-making processes. Negative stimuli often elicit stronger physiological reactions and attract attention more readily, serving as an adaptive mechanism that enables individuals to respond effectively to potential dangers or threats. This heightened sensitivity to negative information aids in harm prevention by facilitating the rapid identification of and response to potential risks.

In contrast, positive bias refers to a stronger response to positive stimuli compared to negative stimuli [83]. Positive bias suggests that in low-arousal or non-threatening situations, humans tend to focus on positive stimuli, fostering exploratory and growth-oriented behaviors. This tendency plays a crucial role in facilitating social interaction, resource acquisition, and learning. By emphasizing positive stimuli in neutral contexts, individuals are more likely to pursue rewarding experiences, build social connections, and explore new opportunities—contributing significantly to overall well-being and survival [79].

These emotional biases are thought to stem from the activation of distinct motivational systems that have evolved to support adaptive behavior. Negative bias is linked to the aversive motivational system, which drives defensive actions such as avoiding danger, thereby ensuring immediate safety and survival [79].

This system triggers physiological and cognitive processes that enable individuals to respond effectively to threats, including heightened vigilance, narrowed attentional focus, and activation of the fight-or-flight response. The aversive motivational system plays a vital role in addressing challenges that directly threaten an individual’s physical or psychological well-being [79].

Positive bias is associated with the appetitive motivational system, which drives approach behaviors like food seeking and building social connections. [79,84,85]. This system encourages actions that lead to long-term benefits, such as building relationships, acquiring resources, and pursuing personal growth. It also stimulates curiosity, exploration, and motivation to achieve goals that enhance one’s quality of life. By promoting approach behaviors, the appetitive motivational system facilitates adaptive functioning in non-threatening environments, allowing individuals to seize opportunities for growth and development [79].

Emotional biases also influence attitudes towards meat consumption in everyday life. Negative bias may lead individuals to focus more intensely on the negative effects of meat consumption, such as environmental pollution, animal welfare, and health concerns. These negative messages can trigger strong emotional reactions, causing some consumers to reduce or avoid meat consumption to prevent these adverse effects. On the other hand, positive bias drives individuals to focus more on the positive aspects of meat consumption, such as taste and nutritional value. In situations without direct threats, positive bias may encourage people to continue enjoying the pleasures of meat, especially in social settings or special occasions where meat often symbolizes abundance and celebration [86,87].

Thus, emotional biases play a significant role in shaping our attitudes and behaviors toward meat consumption, influencing how we balance the potential benefits and risks of eating meat. Understanding these biases can help create more effective dietary interventions that help individuals find a balance between enjoying food and considering health and environmental concerns.

In conclusion, emotional biases, such as negative bias and positive bias, play fundamental roles in human behavior by guiding adaptive responses to different types of stimuli. These biases are crucial for the functioning of motivational systems that support survival and well-being, helping humans navigate a world filled with challenges and opportunities.

#### 2.7.2. Pompian’s Emotional Biases

Pompian [80] identified several emotional biases that influence decision-making. These biases can significantly impact consumer behavior, including choices related to meat consumption.

Endowment bias arises when individuals assign greater value to items that they own compared to those that they do not own. In the context of meat consumption, this bias may lead consumers to overvalue their current meat products or dietary habits, making them resistant to change. Such overvaluation creates a barrier to adopting new dietary practices, including reducing meat consumption or transitioning to plant-based alternatives [80].

Self-control bias refers to the tendency of individuals to prioritize short-term consumption desires over long-term goals, such as improving their health and promoting environmental sustainability. In the context of meat consumption, this bias manifests as difficulty resisting the immediate appeal of meat dishes, despite the well-documented benefits of reducing meat intake for both one’s personal health and the environment. The pursuit of instant gratification often takes precedence over consideration of long-term advantages.

Regret aversion bias is the inclination to steer clear of decisions or actions that could lead to feelings of regret in the future [80]. In the context of dietary habits, consumers may fear dissatisfaction after making changes to their diet, discouraging them from modifying their habitual meat consumption or exploring healthier options. This fear-driven reluctance to change poses a significant barrier to adopting more sustainable eating practices.

Optimism bias describes the tendency for individuals to underestimate the likelihood of negative events while maintaining an overly positive outlook on future outcomes [80]. Regarding meat consumption, this bias may lead consumers to downplay the potential health or environmental consequences of excessive meat intake, delaying efforts to reduce their consumption. This perception fosters complacency, as individuals assume that negative outcomes are unlikely to impact them personally.

Status quo bias refers to the inclination to maintain the current situation rather than embrace changes, as changes often involve uncertainty and potential risks [80]. In the context of meat consumption, this bias leads consumers to stick to their existing dietary habits and resist adopting plant-based alternatives, even when these alternatives are healthier. Since meat consumption has become a part of daily life, advocating for reduced meat intake is challenging, because most people find it easy to accept the notion that “things have always been this way” and are reluctant to make changes. Status quo bias also promotes behavioral inertia, making people less likely to change even when presented with better alternatives such as vegetarian or plant-based protein options. Understanding this bias can help develop strategies like small changes or nudges to guide people towards dietary adjustments without requiring drastic changes.

These aforementioned emotional biases may influence consumers’ meat choices, hindering the transition to more sustainable and health-conscious diets. One potential scenario is that these emotional biases may prevent individuals from fully engaging with VR films, thus failing to produce significant intervention effects. Those with higher levels of empathy are relatively less affected by emotional biases, enabling them to immerse themselves in VR settings and consequently achieve significant intervention outcomes.

### 2.8. Culture and Meat Consumption

Meat consumption is not merely a dietary choice; it is deeply embedded within the cultural, social, and economic fabric of societies across the globe. While dietary habits are often shaped by individual preferences, they are profoundly influenced by broader cultural and societal values. Understanding the intersection of culture and meat consumption is crucial for addressing global challenges such as sustainability and public health. By examining examples from Europe and East Asia, this study seeks to uncover the cultural dynamics that might possibly influence meat consumption. Below is an exploration of how cultural factors influence meat consumption in various countries.

In Denmark, meat consumption is deeply shaped by social values, where meat is often associated with wealth and a high quality of life [86]. For many Danes, consuming meat represents a display of social status and affluence, making it a central element of the Danish diet. Although health concerns may prompt some individuals to consider reducing their meat consumption, these efforts are frequently overshadowed by the dominant cultural and social values [86].

In Sweden, cultural identity and social belonging play a significant role in shaping dietary habits. According to Collier et al., (2021), meat consumption in Sweden exhibits fluctuating trends, partly driven by a skepticism toward meat substitutes. The traditional cultural identity in Sweden reinforces adherence to existing dietary practices, creating barriers to efforts aimed at reducing meat consumption [88].

In Scotland, meat consumption is strongly influenced by social and cultural values. Research by Macdiarmid, Douglas, and Campbell (2016) highlights that for many Scots, meat represents more than just food—it is a lifestyle symbol closely associated with enjoyment, social interactions, and traditional values [87]. These cultural influences pose substantial challenges to initiatives aimed at reducing meat consumption, particularly in settings that prioritize social and familial connections.

Unlike Europe, East Asia has undergone substantial shifts in its meat consumption culture over the past few decades, largely driven by economic growth and globalization. Historically, East Asian countries relied predominantly on grains and vegetables, with religious and cultural norms limiting meat consumption. However, rising incomes and the influence of Western dietary habits have increasingly made meat products a central component of daily diets in the region [89].

In China, the meat consumption culture has transformed significantly with economic growth. Once primarily used as a condiment, meat has become a central element of daily meals and is now regarded as a symbol of an enhanced quality of life [89].

In Korea, meat consumption has been shaped by Mongolian cultural influences, resulting in a variety of cooking styles that are widely celebrated [90]. Meat plays a pivotal role in Korean culinary traditions, especially during family and social gatherings. In Japan, the influence of Western dietary culture is evident in the “Westernization” of meat consumption, reflecting significant shifts toward Western-inspired eating habits [91].

Overall, cultural and social factors might play a significant moderating role in meat consumption habits across countries. These cultural contexts not only shape people’s attitudes toward meat but also strongly influence the process of behavioral change aimed at reducing meat consumption. In Europe, cultural identity and social values often hinder the effectiveness of behavior change in certain contexts. In East Asia, economic development, cultural identity, and Western influences interact to produce diverse responses to advocacy for reducing meat consumption.

To effectively promote policies and initiatives aimed at reducing meat consumption in these regions, it is essential to fully understand the moderating role of cultural factors. Tailored strategies should be designed to accommodate different cultural contexts and social groups, ensuring more effective attitude and behavior changes. Although this study does not specifically emphasize cultural factors, related analyses provide important contextual references for understanding the relationship between attitudes and behaviors toward meat consumption.

## 3. Research Hypotheses

In our research, we examine whether an individual’s ability to feel empathy will predict the sense of presence in a “belief in eating beef” case study. Moreover, the sense of presence will change the attitude toward meat consumption. Based on the literature review, we formulate the following hypotheses:

**Hypothesis 1** **(H1):** *Participants who are more empathic will feel more present in virtual reality.*

**Hypothesis 2** **(H2):** *After watching VR, the higher the level of presence is, the lower the future beef consumption tendency will be.*

Although Nicovich et al., proposed a similar hypothesis to H1, which was supported, their study focused on flight simulation, primarily emphasizing skill-based interaction, where the content of the experience was more functional [21]. This study, however, explores a comparable mechanism in a different virtual context to test whether the influence of empathy on presence is applicable across varied scenarios.

Furthermore, based on the literature, our study considers two mediators of behavior change. First, based on Section 2.5, we examine the change in the perception that cows suffer, which is viewed as the empathy induced by the intervention. Second, based on Section 2.3, we examine the change in anti-beef-eating attitudes, which represents the attitudinal shift prompted by the intervention. These mediators are crucial for understanding how the intervention influences participants’ behaviors toward beef consumption, highlighting the psychological shifts that underpin these changes.

**Hypothesis 3a** **(H3a):** *The relationship described in H2 is mediated by participants’ increased anti-beef-eating attitude, which in turn leads to a decrease in future beef consumption tendencies.*

**Hypothesis 3b** **(H3b):** *The relationship described in H2 is mediated through establishing attitudes toward the suffering of cows, which in turn leads to a decrease in future beef consumption tendencies.*

To strengthen our arguments and validate the critical role of presence, we compare the future beef consumption tendencies among the VR group with a high level of presence, VR group with a low level of presence, and the video group. Since we do not know the difference in the intervention effects between the VR with a high level of presence and the video group, we will not make assumptions about the two. We make the following hypothesis:

**Hypothesis 4** **(H4):** *Regarding the future beef consumption tendency after the intervention, the VR group with a high level of presence will show a lower tendency than the VR group with a low level of presence.*

**Figure 1:**
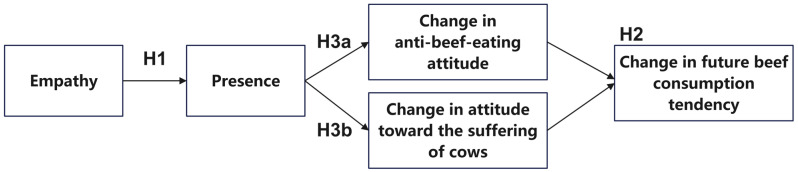
A conceptual model of the primary hypotheses for empathy, presence, and attitudes toward beef consumption.

Our primary hypotheses for empathy, presence, and attitudes toward beef consumption are formulated as a conceptual model (Figure 1). The hypotheses are tested in the following sections.

## 4. Methodology

### 4.1. Research Design

This study incorporated an experimental condition and a control group: a VR condition and a video condition. Before the experiment, participants completed several questionnaires covering their experiences in virtual environments, attitudes toward the suffering of cows, and meat consumption habits. In the VR group, we used the Meta Quest 3 VR headset, dual hand-held controllers, and a computer equipped with an ASUS Dual RTX 4070 O12G graphics card as the setup. The VR content was created using Unreal software, version 5.3. The laboratory space was 2.5 × 2.5 m in size. Participants could change their view by turning their heads, and since each participant held a controller in both hands, they could control the virtual body’s hands and movements using the controllers.

The virtual reality content featured a virtual farm with two cows and one injured cow lying on a table. The VR experience lasted approximately 4 min. Participants could move around the farm but were informed that the cow on the table was injured.

Participants wore VR headsets to enter the virtual world. Initially, they were instructed to explore their surroundings by interacting with elements, such as touching the cows’ heads, which triggered auditory responses from the cows (participants could see their virtual hands and control them using the controllers). As they navigated the environment, a pre-recorded narration described the life of a cow. They were then directed to approach a table where an injured cow lay, and the pre-recorded narrative informed them that the cow was hurt and struggling, enabling them to engage with the scenario through a first-person viewpoint. After exiting the VR scenario, participants completed the initial questionnaires again.

Participants in the video condition watched a video on a desktop computer with a 22-inch LCD screen at a resolution of 1920 × 1080. The video’s visual and auditory content matched that of the VR scenario, including the same narration about the life of a cow. However, unlike the VR condition, participants in the video group had a passive role; they were unable to manipulate the mouse or keyboard to adjust the video’s speed or angle and could only watch the video without interacting with the content. The perspective was first-person, but participants did not see themselves in the video.

### 4.2. Participants

This study was approved by the Research Ethics Committee of National Taiwan University and classified as a full board review on 18 July 2023. The Research Ethics Committee approval number is NTU-REC No.: 203305HM005.

Participants were primarily recruited through social networking sites (SNSs). As a token of appreciation for their participation, participants were provided with a financial incentive. Our study aimed to investigate the effects of VR and empathy on attitudes toward meat consumption. Therefore, we specifically recruited participants who were non-vegetarians, as the focus was on attitudes related to meat consumption. Vegetarians were excluded to ensure that participants’ baseline dietary habits would not bias the findings.

Furthermore, due to the potential for motion sickness caused by the VR headset, we also excluded individuals who had previously experienced dizziness or discomfort from using VR devices. This precaution was taken to avoid any adverse physical reactions that could interfere with participants’ engagement in the VR experience and potentially affect the results.

A power analysis was conducted to determine the required number of participants for this study. Specifically, for hypothesis H4, which involves a three-group ANOVA, G*Power was utilized to estimate the necessary sample size. To achieve a statistical power of 0.8 with an alpha level of 0.05, and assuming a medium to large effect size (f ranging from 0.25 to 0.4), a total of 66 to 159 participants are needed.

A total of 142 participants (50.00% female; mean age [M] = 25.22; standard deviation [SD] = 8.18) completed the study, with 77 participants in the VR condition and 65 in the video condition. Additionally, based on the measured levels of presence, the VR condition was subdivided into two groups: 44 participants in the high-level-of-presence VR group and 33 participants in the low-level-of-presence VR group. In addition, the gender composition ($\chi^{2}\left(2\right)=0.137;\;p=0.91$) and age (*F*(2, 139) = 2.499; *p* = 0.082) are not significantly different in these three groups, indicating that although we did not place any constraints on the recruitment of the participants, our analyses may exclude potential confounding characteristics.

### 4.3. Measurement

In this research design, participants were randomly assigned to one of two conditions: the virtual reality (VR) condition or the video condition. Prior to the experiment, participants completed a series of questionnaires covering demographic information, the empathy scale, beef consumption, attitudes toward beef consumption, and attitudes toward the suffering of cows. The questions regarding attitudes toward beef consumption and the suffering of cows were administered both before and after the experiment.

To measure empathy, the study utilized the empathy scale developed by Tian and Robertson (2019), which includes items such as “I sometimes try to understand my friends better by imagining how things look from their perspective”. The scale consists of 10 items, with responses recorded on a 7-point Likert scale ranging from 1 (Strongly disagree) to 7 (Strongly agree) (α = 0.71) [48].

The study also incorporated revised versions of Anderson et al.’s questionnaires to assess beef consumption and related attitudes. For future beef consumption tendency, participants respond to the following question: “Thinking about your diet in the next 7 days, how often will you eat meals (including breakfast, lunch, dinner, etc.) or snacks that contain any type of beef (ribs, steak, strip, beef jerky, etc.)?” The response options are as follows: (1) Never, (2) 1–3 times per month, (3) 1 time per week, (4) 2–4 times per week, (5) 5–6 times per week, and (6) 1 or more times per day [19]. The other question is “In the coming week (21 meals), how many meals would you want to eat beef or beef-related products?” The participants fill in the number of meals. The Cronbach’s coefficient was 0.76.

Regarding attitudes toward beef consumption, participants answer questions such as “It is important to minimize the amount of beef (ribs, steak, strip, beef jerky, etc.) a person consumes” and “It is important for everyone to reduce their consumption of beef” (α = 0.76). Responses are recorded on a 5-point scale ranging from “Strongly disagree” to “Strongly agree”.

For attitudes toward the suffering of cows, participants are asked questions like “Eating beef (ribs, steak, strip, beef jerky, etc.) directly contributes to the suffering of cows” (α = 0.62). Responses for these items are also recorded on a 5-point scale ranging from “Strongly disagree” to “Strongly agree”.

To assess whether varying levels of immersive media experiences lead to differences in participants’ sense of presence, we employed the Spatial Presence questionnaire, which boasts high reliability (α = 0.93) and was adapted from Tian et al., (2004). The questionnaire was a multi-faceted measurement based on Spatial Presence theory, with confirmed reliability and validity. In this study, to measure the sense of “self-location”, participants were instructed to rate their agreement with items on the “Spatial Presence: Self Location” (SPSL) subscale using a 7-point Likert scale, where 1 indicated “strongly disagree” and 7 indicated “strongly agree”. Example statements included, “I felt like I was really on the farm”, “I felt like I was part of the virtual environment”, “I felt like the objects in the scene surrounded me”, and “I felt like I was part of the environment depicted in the scene”. These items were designed to measure the extent to which participants experienced a sense of being physically present within the virtual environment [48].

### 4.4. Statistical Analysis

A Pearson correlation analysis was performed to explore the relationships between variables, with a specific focus on verifying the correlation between scores on the empathy scale and VR presence (H1). This initial analysis helped identify the direction and strength of associations among the variables and also helped eliminate potential concerns of reverse causation.

Second, to examine whether a high level of presence in VR may influence future beef consumption tendencies by affecting participants’ attitudes toward beef consumption and perceptions of the suffering of cattle (H2, H3a, H3b), a mediation model was employed. Path analyses tested the total effect (H2) and the indirect pathway of “Presence → Attitudes Toward Beef Consumption → Future Beef Consumption Tendency” to see if attitudes act as a significant mediator (H3a). Additionally, this step evaluated if perceptions of the suffering of cattle play a significant mediating role in this model (H3b). Significant findings in these indirect pathways would support the hypotheses, helping clarify the mechanisms by which VR presence impacts beef consumption attitudes and intentions.

Third, a one-way ANOVA was conducted to evaluate the effects of different levels of VR presence (high and low) on future beef consumption tendencies (H4). To include the video group for comparison, the VR group was divided into two groups using the mean score of their levels of presence as a threshold. This analysis compared pretest and post-test changes in future beef consumption tendencies across the high-level-of-presence VR group, low-level-of-presence VR group, and video group. If the ANOVA yielded significant results, a post hoc Tukey HSD test was performed to identify specific differences between the groups. To ensure the validity of the ANOVA analysis, we also used an ANOVA and chi-squared test to ensure that the three groups comprised subjects with similar distributions of gender and age. This approach helped determine whether a high level of presence in the VR scenario is associated with a stronger reduction in beef consumption intentions than in the other groups.

Finally, the significance level for this study was set at *p* < 0.05 for hypothesis testing. Any significant results at each stage of analysis were treated as evidence supporting the relevant hypotheses.

## 5. Results

The descriptive statistics are presented in Table 1. The score of empathy scale measured in the pretest is significantly correlated with the level of presence in VR: *r* = 0.335; *p* = 0.003. The results support H1 and not the reverse direction, because the empathy scale was measured before the VR scenario. This temporal ordering clarifies that the levels of empathy were established prior to participants’ exposure to the VR experience.

For H2, H3a, and H3b, it was hypothesized that higher engagement in a VR setting would decrease participants’ willingness to consume beef, increase their perception of the suffering of cattle, and ultimately reduce their future beef consumption tendencies. The total effect of this mediation model was significant (β=−0.196; p=0.008) with only the path through attitudes toward beef consumption showing a significant indirect effect: β=−0.033; p=0.029. The direct effect of presence negatively predicting future beef consumption tendency was also significant: β=−0.172; p=0.020. This indicates that participants who are engaged in the VR setting are more likely to oppose decisions to eat beef, which in turn reduces their own willingness to consume beef. However, this effect was not mediated by attitudes toward the suffering of cows, indicating that the reduction in intentions to consume beef is not primarily influenced by the empathy elicited during the intervention.

The results from the one-way ANOVA suggest that there is a significant difference in the change in future beef consumption tendency between the pretest and post-test results across the high-level-of-presence VR group, the low-level-of-presence VR group, and the video group, with *F*(2, 139) = 4.585 and *p* = 0.012. The post hoc Tukey HSD test further indicates that the change in future beef consumption tendency in the low-level-of-presence VR group is smaller than that observed in both the high-level-of-presence VR group and the video group, *p* = 0.021. This finding supports H4, demonstrating that the intervention effect of VR on reducing future beef consumption is only comparable to that of the video intervention when the level of presence within the VR environment is high. The gender ($\chi^{2}\left(2\right)=0.137;\;p=0.91$) and age (*F*(2, 139) = 2.499; *p* = 0.082) compositions are not significantly different in these three groups, indicating that the three groups are composed of participants with similar characteristics.

## 6. Discussion

The findings of this study underscore the complex interplay between empathy, presence, and the impact of virtual reality interventions on attitudes toward beef consumption. Our results support Hypothesis 1, indicating that participants with higher levels of empathy experienced a greater sense of presence in VR, which enhanced the immersive experience and potentially made the interventions more effective. However, the direct and indirect effects observed in Hypotheses 2, 3a and 3b suggest that while the level of presence in VR can directly reduce future beef consumption tendencies, this effect is not mediated by attitudes toward the suffering of cows. This implies that the sense of presence in VR may reduce beef consumption through mechanisms other than increased empathy for the suffering of cows.

Moreover, the one-way ANOVA analysis supporting Hypothesis 4 demonstrates that the efficacy of VR interventions in altering beef consumption behavior depends on the level of presence experienced. This suggests that simply using VR technology is not sufficient to change behavior; the degree of presence and the participant’s empathy are crucial factors. Collectively, these results highlight the potential of VR as a tool for behavior change, contingent on the participant’s level of empathy.

Furthermore, our research findings suggest that changes in empathy—specifically regarding attitudes toward the suffering of cows—observed during the experiment may not be as impactful as participants’ inherent empathy levels. This implies that a participant’s existing empathy plays a significant role in the effectiveness of VR interventions. This nuanced understanding of empathy underscores its significance not only as an individual characteristic but also as an essential element for successful communication within virtual environments.

These findings suggest that VR can serve as a powerful tool for environmental and ethical advocacy, with the potential to significantly alter dietary habits and attitudes toward animal welfare and sustainability. However, VR’s effectiveness may be diminished when participants exhibit low levels of empathy and presence. In such cases, traditional video viewing proves more effective in influencing meat consumption behaviors. This indicates that while VR holds substantial potential, its impact depends on the participant’s sense of presence and emotional investment, both of which are critical to realizing the full benefits of immersive virtual experiences.

Incorporating our findings, we conclude that VR interventions can effectively influence consumer attitudes toward meat (beef) consumption, reduce meat intake, and, in turn, help prevent cancers and cardiovascular diseases.

## 7. Conclusions

Our study engages in a rich dialog with previous research by using virtual reality (VR) to explore how empathy, as a psychological mechanism, influences attitudes toward meat consumption. Many earlier studies, such as those by Herrera et al., (2018) and Bujić et al., (2020), have shown that VR can enhance users’ empathic responses, thereby promoting prosocial behaviors [12,49]. However, these studies mainly emphasize VR’s role in enhancing empathy to influence behavior, without delving into how empathy, as a stable trait, may foster a stronger sense of presence in VR experiences, further affecting attitudes and behaviors.

Our findings further reveal that, in a VR setting, individuals with higher empathy scores experience a stronger sense of presence, which significantly influences their attitudes toward beef consumption. Unlike previous studies that assume that presence precedes empathy, our results support the opposite view: empathy may be the key factor triggering a heightened sense of presence. This aligns with theories proposed by Nicovich et al., (2005) and Lombard and Ditton (1997), which highlight the importance of an individual’s ability to feel empathy in digital media interactions [21,69].

Additionally, compared to some studies, such as those by Schutte and Stilinović (2017) and Barbot and Kaufman (2020), which emphasize VR as the “ultimate empathy machine”, our study highlights the interactive effect of individual differences on empathy and presence [14,40]. This implies that considering individual empathy levels may be more crucial when designing VR interventions to promote healthy dietary habits. Such adjustments could enhance the effectiveness of VR interventions, making them more impactful in fostering healthy eating behaviors.

In summary, our research not only demonstrates VR’s potential as an effective intervention tool but also underscores the importance of incorporating empathy as an individual trait when designing VR intervention strategies to achieve long-term behavior change and public health improvement.

## 8. Limitations, Suggestions, and Practical Implications

This study suggests that VR could play a critical role in promoting healthier dietary choices. However, our study, which recruited participants solely in Taiwan, has some limitations regarding the generalizability of the findings, particularly because dietary habits can vary significantly across different cultural contexts. Consequently, our results may not be fully applicable outside of Taiwan. Furthermore, our reliance on self-reported questionnaires may have introduced bias into the data.

In designing the study, we aimed not only to assess the immediate impact of our intervention on attitudes toward meat consumption but also to determine if these changes would be sustained over time. To address this, participants completed a questionnaire on their meat consumption habits and related attitudes before and immediately after the intervention, allowing us to gauge any short-term effects.

To further explore the long-term impact, we followed up two weeks post-intervention by inviting participants to complete the same questionnaire again. Unfortunately, fewer than 10 out of 142 participants responded to this follow-up, which provided an insufficient sample for meaningful analysis and thus lacked representativeness. As a result, we excluded these data from our study findings, acknowledging this as a limitation.

We believe that understanding the long-term sustainability of these changes is crucial for designing effective interventions. Evaluating the long-term effects of VR-based interventions helps assess the lasting impact of these tools and confirms their value in driving significant attitude changes.

In light of this, we suggest that future research use more effective engagement strategies to increase response rates in follow-up studies, yielding more reliable long-term data. Personalized communication, frequent reminders, and incentives for participation could help overcome challenges associated with low response rates. For instance, participants could be offered rewards, such as gift cards or entries into a prize draw, as an incentive to complete follow-up questionnaires. Clear communication about the importance of follow-up data for the study’s success could also enhance participants’ motivation and commitment.

Another important strategy is incorporating qualitative methods, such as in-depth interviews and focus group discussions, to obtain insights into how participants perceive, adapt to, and internalize new attitudes and behaviors over time. These qualitative approaches provide rich, detailed data on participants’ experiences, helping researchers understand the nuanced processes underlying attitude change. By understanding participants’ personal motivations, perceived challenges, and emotional and cognitive responses to the intervention, researchers can develop more tailored and effective VR interventions.

Combining quantitative data with qualitative insights could provide a more comprehensive evaluation of the intervention’s impact. While quantitative data, such as survey responses, measure the extent of change, qualitative insights shed light on the mechanisms of change, barriers encountered, and participants’ emotional journeys. This mixed-methods approach strengthens the robustness of the findings and helps in tailoring future interventions for a greater and more lasting impact.

By integrating these strategies, future research can enhance our understanding of how VR interventions bring about sustained changes in attitudes. Such efforts are crucial for refining VR as an effective tool for promoting healthier, more sustainable lifestyle choices, ultimately contributing to public health and environmental sustainability.

The use of a unified empathy measurement in this study may obscure the distinct effects of cognitive and affective empathy. Furthermore, the study does not directly compare the outcomes of integrated and separate empathy measurements, which limits its ability to address the specific advantages or disadvantages of each approach. Furthermore, the current measurement strategy has limited capacity to identify the unique impacts of cognitive or affective empathy in VR settings, potentially overlooking critical nuances in these environments.

Future research comparing the effects of integrated and separate empathy measurements is essential to refine our understanding of different aspects of empathy and its applications in virtual environments. Conducting comparative analyses of both approaches will provide valuable insights, enabling researchers to select the most appropriate tools based on their research goals. These studies should explore the differences between integrated and separate measurements, offering deeper insights into their respective strengths and limitations. In addition, investigating the specific effects of cognitive and affective empathy in VR contexts will help uncover their unique roles. Developing hybrid measurement tools that balance efficiency and specificity could further advance empathy research. Finally, expanding research to include diverse VR applications could enhance our understanding of empathy’s role across various scenarios, contributing to more effective VR-based interventions.

On top of that, in the current literature, research on the influence of emotional biases on meat consumption remains relatively limited. According to our literature review, Pompian [80] and Yuan et al.’s [79] studies are among the few studies that have systematically explored emotional biases and their impact on consumer decision-making. Therefore, we have chosen to base our study on the findings of Pompian and Yuan et al. [79,80] and to hypothesize, based on their conclusions, the potential influence of emotional biases on meat consumption choices. This study acknowledges that one of its limitations is the lack of comprehensive research available in the current literature. Future research could further expand on this area to validate these hypotheses and address the existing knowledge gaps.

Cultural and social values may exert indirect influence on the processes of attitude formation and behavior expression. While these factors are not the core focus of this study, their discussion contributes to supplementing the understanding of attitude and behavior changes in meat consumption. However, the limited scope of this study means that cultural factors were not explored in depth, which may affect the generalizability of its findings across different cultural contexts. Future research that incorporates the moderating role of cultural contexts in the relationship between attitudes and behaviors would more comprehensively support the findings of this study and enhance its academic contribution.

We suggest that future research could delve deeper into analyzing the moderating role of cultural and emotional biases to further enhance the generalizability of the findings and address the identified limitations.

Another limitation of our study is that we did not explore adjustments to the VR intervention for audiences with varying sensitivities to animal suffering. Different audiences may respond differently to such content, which could impact the intervention’s effectiveness. Addressing this issue in future research could involve tailoring VR experiences to meet the needs of different audiences and evaluating the sustained effects of these interventions over time. This would help make VR interventions more targeted and effective in fostering attitudinal change.

To translate our research into feasible strategies for long-term practice, several approaches can be considered. First, sustained interventions involving repeated VR experiences could help participants continuously experience the impact of empathy, potentially leading to lasting behavioral changes. These experiences could be progressively staged, beginning with basic knowledge about animal life and gradually moving to deeper emotional scenarios. Second, integrating VR empathy experiences into social influence programs—such as collaborations with health organizations or environmental groups—could broaden the impact. Such partnerships could help incorporate VR into public events, campaigns, and educational initiatives, creating a wider social effect. Lastly, policy advocacy could promote the integration of VR empathy experiences into education, health, and environmental protection sectors, potentially requiring schools or companies to include these interventions in their health initiatives.

By using these strategies, the findings of our research on empathy and VR can extend beyond immediate effects, contributing to sustained behavioral changes through education, social activities, and policy support. Ultimately, this could promote public health and improve societal attitudes toward the environment and animals.

## Figures and Tables

**Table 1:** Descriptive statistics.

Variable	Mean	SD	1	2	3	4	5	6	7	8
1. Empathy scale	3.658	0.479	—							
2. Pretest FBCT	3.162	3.170	−0.143	—						
3. Pretest ATBC	2.275	0.708	0.178 *	−0.302 ***	—					
4. Pretest ATCS	3.272	0.877	0.165 *	−0.241 **	0.422 ***	—				
5. Presence	4.974	1.558	0.335 *	−0.032	0.376 ***	0.174	—			
6. Post-test FBCT	2.588	3.068	−0.144	0.902 ***	−0.371 ***	−0.314 ***	−0.166	—		
7. Post-test ATBC	2.775	0.930	0.108 *	−0.212 *	0.750 ***	0.458 ***	0.426 ***	−0.311 ***	—	
8. Post-test ATCS	3.582	0.939	0.115 *	−0.176 *	0.392 ***	0.701 ***	0.358 **	−0.276 ***	0.604 ***	—

Notes: FBCT = future beef consumption tendency. ATBC = attitudes toward beef consumption. ATCS = attitudes toward the suffering of cows. For variables other than presence, *n* = 142. For presence, *n* = 77. * *p* < 0.05, ** *p* < 0.01, *** *p* < 0.001.

## Data Availability

The original contributions presented in the study are included in the article, further inquiries can be directed to the corresponding author.
